# Population-based study of ovarian cancer in Côte d'Or: prognostic factors and trends in relative survival rates over the last 20 years

**DOI:** 10.1186/1471-2407-10-622

**Published:** 2010-11-10

**Authors:** Zeinab Hamidou, Sylvain Causeret, Tienhan S Dabakuyo, Julie Gentil, Laurent Arnould, Patrick Roignot, Thierry Altwegg, Marie-Laure Poillot, Franck Bonnetain, Patrick Arveux

**Affiliations:** 1 Registre des Cancers du Sein et autres Cancers Gynécologiques de Côte d'Or, Centre Georges François Leclerc, Dijon, France; 2 EA 4184, Université de Bourgogne, Dijon 21000, France; 3 Centre de Pathologie, 33 rue Nicolas Bornier, 21000, Dijon, France; 4 Centre de Radiothérapie Du Parc, 18 cours du général de Gaulle, 21000 Dijon, France; 5 Unité de Biostatistiques et d'épidémiologie, Centre Georges François Leclerc, Dijon Cedex 21079, France

## Abstract

**Background:**

The aim of this population-based study was to assess independent prognostic factors in ovarian cancer using relative survival (RS) and to investigate changes in RS rates from 1982 to 2005.

**Methods:**

Data on 748 patients with ovarian cancer were provided by the Côte d'Or gynaecologic cancer registry. The RS was estimated using a generalized linear model with a Poisson error structure. Relative survival and its 95% confidence interval (CI) were described at the following specific time points 1, 3 and 5 years. The effect of prognostic factors on survival was assessed with multivariate analyses of RS.

**Results:**

The median follow-up was 12 years. The RS rates at 1, 3 and 5 years were 81%, 55% and 44%, respectively. As compared with the period 1982-1989, an improvement in survival was found for the period 1998-2005: HR = 0.52[0.40-0.67]. Women who lived in urban areas had better RS: HR = 0.82[0.67-0.99]. Patients with epithelial types of ovarian cancer other than mucinous or endometrioid cancer had worse RS than those with serous histology. Age ≥ 70 years was associated with lower survival.

**Conclusions:**

Period of diagnosis, stage at diagnosis, histology, place of residence and age were independent prognostic factors for survival in ovarian cancer. An improvement in the survival rate was observed after 1998 but a significant improvement was limited to advanced stage cancers.

## Background

Ovarian cancer is the sixth most common cancer in women. Incidence rates are highest in developed countries [1]. In France, ovarian cancer accounted for 4375 new cases and 3180 deaths in 2005 [2].

Despite improved knowledge of prognostic factors and advances in treatments for ovarian cancer, the outcome in terms of long-term survival remains unknown. In fact, while some studies [3,4] showed a significant increase in the survival rate over time, others showed similar survival rates over time [5]. Moreover, many previous studies on prognostic factors and survival in ovarian cancer were based on selected patients, and did not use relative survival (RS) as the primary end-point [4,6-8].

To our knowledge, no population-based study has used RS as the primary end-point to determine independent prognostic factors in ovarian cancer. Relative survival is an estimator of the excess mortality ratio. The use of RS could make it possible to correct indirectly for deaths not attributable to ovarian cancer [9].

The Côte d'Or gynaecologic cancer registry is the only cancer registry in France that focuses on gynaecologic cancers. It has been collecting comprehensive population-based data since 1982.

Our purpose was to study relative survival according to the main patient and tumour characteristics.

## Methods

### Population

Patients with ovarian cancer were identified from the Côte d'Or gynaecologic cancer registry. This study included all patients diagnosed with primary ovarian cancer from January 1982 to December 2005. Ethical approval from the national ethics committee (Commission Nationale de l'Informatique et des Libertés) was obtained for the Côte d'Or gynaecologic cancer registry, which allowed the use of data recorded in this registry for medical studies. Written informed consent was also obtained from every participant at their first medical examination.

### Studied variables and endpoints

Demographic data obtained from each patient's medical records included age at diagnosis and menopausal status. The town of residence was also known for each patient and was defined as a rural or urban area using data provided by the National Institute for Statistics and Economic Studies (INSEE) of France. We also obtained clinical data on the histological type and the surgical stage according to the International Federation of Gynecology and Obstetrics (FIGO) system; staging was based on clinical information when the surgical stage was not available. Patients were categorised into three age groups: < 50 years, 50-70 years, and ≥ 70 years. For the histological type, cases were divided into four groups: serous, mucinous and endometrioid, other epithelial types and non-epithelial tumours. The majority of the other-epithelial group were clear cell tumours and unspecified adenocarcinomas, whereas the non-epithelial group included germ cell and sex-cord stromal tumours. Years of diagnosis were grouped into 3 periods: 1982-1989, 1990-1997 and 1998-2005.

Survival was estimated from the date of diagnosis until the date of death (all causes). Patients who were alive at the cut-off date or died after the cut-off date (01 January 2009) were censored.

### Statistical methods

Continuous variables are described as means, standard deviations (SD), and medians, and qualitative variables are given as percentages. The percentage of missing values is also provided. Follow- up was calculated using the reverse Kaplan Meier method which is calculated in the same way as the Kaplan-Meier estimate of survival function, but with the meaning of the status indicators reversed [10].

Relative survival (RS) is the ratio between the observed survival rate in the patient group and the expected survival rate of a comparable group from the general population of the same age, sex and year of diagnosis. Relative survival represents an estimation of survival eliminating the effect of competing causes of death. Annual mortality rates for the entire female population of Côte d'Or stratified by age and calendar time were used to calculate expected survival. The RS was estimated using a generalized linear model with a Poisson error structure [9].

Relative survival and its 95% confidence interval (CI) were described at the following specific time points 1, 3 and 5 years. Univariate and multivariate relative survival analyses were performed to determine independent prognostic factors in ovarian cancer. Five-year RS estimates and their 95% CIs were calculated for variables of interest across the study periods and according to the stage of diagnosis.

All tests were two-tailed and the level of p < 0.05 was considered statistically significant. Analyses were performed using Statistical Analysis Software version 9.1 and STATA (version 10).

## Results

### Population

A total of 808 women were diagnosed with ovarian carcinoma from 1982 to 2005. Among them, 60 were lost to follow-up at the date of diagnosis. Thus, a total of 748 were retained for the study. The demographic and clinical characteristics of the study population are provided in Table 1 and the distribution of these factors over the study periods is shown in Table 2. The median age at diagnosis was 62 years (range, 11 to 94 years) and the mean age was 61 years (SD = 14.8). Tumour characteristics did not vary, on the whole, over the three periods. However, there was a slight increase in the proportion of patients with stage III disease even though this was not statistically significant (Table 2).

**Table 1:** Patients and tumour characteristics

Variables	Median	Range
**Age (years)**	62	11-94
**Follow-up (years)**	11.9	0.003-26.08
		

	**Number of patients**	**%**

**Age**		
< 50	154	20.6
50-70	359	48.0
≥ 70	235	31.4
		
**Stage at diagnosis**		
I	186	24.8
II	60	8.0
III	387	51.7
IV	90	12.0
Unknown	25	3.3
		
**Histological type**		
Serous	433	58.0
Mucinous	77	10.3
Endometrioid	90	12.0
Clear cell	40	5.3
Adenocarcinoma unspecified	46	6.1
Other epithelial	5	0.6
Sex-cord stromal	30	4.0
Germ cell	27	3.6
		
**Period of diagnosis**		
1982-1989	221	29.5
1990-1997	255	34.1
1998-2005	272	36.4
		
**Place of residence**		
Urban	412	55.2
Rural	335	44.7
Unknown	1	0.1
		
**Menopause**		
Yes	450	60.2
No	138	18.4
Unknown	160	21.4

**Table 2:** Distribution of patients and tumour characteristics by period of diagnosis

	1982-1989	1990-1997	1998-2005	
Variables	Number of patients (%)	Number of patients (%)	Number of patients (%)	Fisher p
	N = 221	N = 255	N = 272	
**Age**				0.722
< 50	44(19.9%)	59 (23.1%)	51(18.7%)	
50-70	110(49.7%)	119(46.7%)	130(47.8%)	
≥ 70	67(30.3%)	77(30.2%)	91(33.5%)	
				
**Stage at diagnosis**				0.091
I	66(29.8%)	58(22.7%)	62(22.8%)	
II	18(8.1%)	27(10.6%)	15(5.5%)	
III	97(43.9%)	134(52.5%)	156(57.3%)	
IV	29(13.1%)	30(11.8%)	31(11.4%)	
Unknown	11(4.9%)	6(2.3%)	8(3.0%)	
				
**Histological type**				
Serous	122(55.2%)	157(61.6%)	154(56.6%)	0.178
Mucinous Endometrioid	51(23.0%)	50(19.6%)	66(24.2%)	
Other epithelial	28(12.7%)	36(14.1%)	27(10.0%)	
Non epithelial	20(9.0%)	12(4.7%)	25(9.1%)	
				
**Place of residence**				
Urban	126(57.0%)	138(54.1%)	148(54.4%)	0.803
Rural	95(42.9%)	116(45.5%)	124(45.6%)	
Unknown		1(0.4%)		
				
**Menopause**				
Yes	160(72.4%)	182(71.4%)	108(39.7%)	0.570
No	43(19.5%)	57(22.3%)	38(14.0%)	
Unknown	18(8.1%)	16(6.3%)	126(46.3%)	

### Relative survival

**Figure 1:**
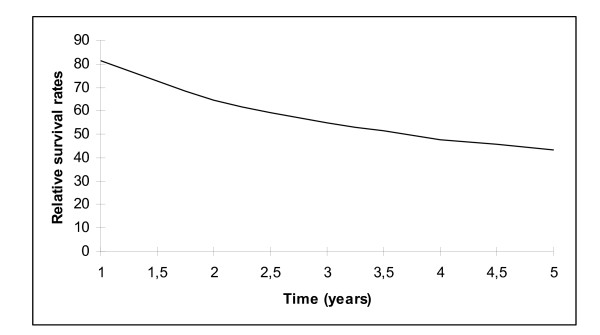
**Relative survival of the study population**.

Median follow up was 11.9 years (range 0.003 to 26 years). At the cut-off date, 516 (69%) patients had died and 56 (7%) were lost to follow-up. The median survival time was 40 months. The RS rates at 1, 3, and 5 years for all patients were respectively 81%, 55% and 44% (Figure1). Table 3 shows RS rates based on patient and tumour characteristics. Five-year RS rates per stage I, II, III and IV were 85%, 48%, 29% and 14%, respectively. Univariate hazard ratios (Table 3) were 4.51[2.69-7.57], 7.99[5.34-11.96] and 12.83[8.18-20.08], for stage II, III and IV, respectively, as compared to stage I. When divided according to age-group < 50 years, 50-70 years and ≥ 70 years, 5-year RS rates were 58%, 43% and 32%, respectively and univariate hazard ratios were 1.51[1.16-1.95] and 2.23[1.78-2.93], respectively, as compared to age-group < 50 years. After dividing our study population into early stage (stage FIGO I and II) and advanced stage disease, table 4 shows that, after controlling for stage, patients aged over 70 years had worse relative survival rates as compared to younger patients (< 50 years) in both the advanced and early-stage group.

**Table 3:** Relative survival according to patient and tumour characteristics: Univariate and multivariate analyses

			Relative survival			Univariate analysis N = 748			Multivariate analysis N = 722		
**Variables**	**Patients at risk**	**Number of deaths**	**1-year RS (CI)**	**3-year RS (CI)**	**5-year RS (CI)**	**HR**	**95% CI**	**P value**	**HR**	**95% CI**	**P value**

**Age**								< 0.0001			0.003
< 50	154	79	89 (82.7-93.0)	68 (60.4-75.3)	58 (50.0-65.9)	1			1		
50-70	359	243	84 (79.6-87.4)	55 (50.8-60.6)	43 (37.8-48.5)	1.51	1.16-1.95	< 0.0001	1.22	0.94-1.59	0.12
≥ 70	235	194	70 (63.4-76.0)	42 (35.2-49.2)	32 (24.9-38.7)	2.23	1.78-2.93	0.001	1.60	1.21-2.12	0.0009
**Stage at diagnosis**								< 0.0001			< 0.0001
I	186	58	98 (93.9-99.3)	89 (82.7-93.1)	85 (78.1-90.0)	1			1		
II	60	44	82 (68.4-89.8)	60.3 (45.9-72.1)	48 (34.5-61.0)	4.51	2.69-7.57	< 0.0001	4.43	2.63-7.46	< 0.0001
III	387	320	78 (73.5-82.0)	44 (38.8-49.1)	29 (24.0-33.6)	7.99	5.34-11.96	< 0.0001	8.80	5.81-13.34	< 0.0001
IV	90	78	62 (50.5-71.1)	28 (18.6-37.8)	14 (6.7-22.7)	12.82	8.18-20.08	< 0.0001	13.07	8.26-20.66	< 0.0001
Unknown	25	16									
**Histological type**								0.0001			0.010
Serous	433	309	84 (80.4-87.7)	56(50.8-60.7)	41 (36.3-46.3)	1			1		
Mucinous- Endometrioid	167	105	82(75.3-87.4)	56 (48.1-63.7)	49 (40.8-56.9)	0.74	0.58-0.96	0.022	1.23	0.95-1.58	0.11
Other epithelial	91	73	65 (54.4-74.4)	40 (29.2-49.8)	31 (21.0-41.2)	1.37	1.04-1.82	0.023	1.62	1.22-2.16	0.0008
Non epithelial	57	29	78 (63.6-87.5)	67 (50.6-78.6)	63 (46.8-75.1)	0.53	0.33-0.84	0.007	1.19	0.72-1.96	0.47
**Period of diagnosis**								0.0004			0.0001
1982-1989	221	159	82 (75.8-86.6)	49 (42.0-56.0)	41 (34.2-48.2)	1			1		
1990-1997	255	191	74 (68.1-79.3)	50 (43.4-56.3)	39 (32.8-45.6)	1.07	0.86-1.34	0.5217	0.96	0.77-1.21	0.79
1998-2005	272	166	87 (82.2-90.8)	64 (57.2-69.3)	49 (42.5-55.5)	0.67	0.52-0.87	0.002	0.52	0.40-0.67	< 0.0001
**Place of residence**											
Rural	335	242	79 (74.0-83.2)	40 (34.7-46.0)	40 (34.7-46.0)	1			1		
Urban	412	273	83 (78.6-86.4)	46 (40.6-51.1)	46 (40.6-51.1)	0.81	0.67-0.99	0.04	0.82	0.67-0.99	0.04
Unknown	1	1									
**Menopause**											
Yes	450	346	78 (74.1-82.2)	49 (43.8-53.6)	36 (31.4-41.0)	1					
No	138	72	88 (81.0-92.5)	70 (60.8-76.7)	60 (50.8-67.7)	1.93	1.48-2.51	< 0.0001			
Unknown	160	98	83 (75.6-88.0)	58 (49.4-65.5)	49 (40.2-57.0)						

**Table 4:** Five-year relative survival rates according to the stage of diagnosis

Variables	N	Deaths	5-year relative survival rates (95% CI)
**Age**			
**FIGO I-II**			
< 50	73	20	80.69 (69.09-88.29)
50-70	111	40	79.50 (69.94-86.32)
>=70	61	42	59.88 (43.12-73.15)
**FIGO III-IV**			
< 50	78	59	35.83 (25.11-46.65)
50-70	231	192	26.31 (20.59-32.36)
> = 70	155	137	20.70 (13.91-28.44)
**Histologic type**			
**FIGO I-II**			
Serous	101	44	73.38 (61.98-81.85)
Mucinous-endometroîd	78	31	74.94 (62.74-83.65)
Other epithelials	34	19	65.82 (45.99-79.83)
Non epithelials	32	8	93.79 (67.65-98.95)
**FIGO III-IV**			
Serous	315	255	31.11 (25.68-36.68)
Mucinous-endometroîd	82	71	21.36 (12.94-31.19)
Other epithelials	49	48	6.21 (1.41-16.34)
Non epithelials	*	*	*
**Period of diagnosis**			
**FIGO I-II**			
1982-1989	84	39	73.44 (61.20-82.35)
1990-1997	84	38	75.40 (63.00-84.15)
1998-2005	77	25	80.83 (67.98-88.93)
**FIGO III-IV**			
1982-1989	122	110	19.46 (12.53-27.52)
1990-1997	160	145	20.31 (14.24-27.15)
1998-2005	182	133	36.24 (28.68-43.84)
**Place of residence**			
**FIGO I-II**			
Urban	143	59	75.02 (65.89-82.03)
rural	102	43	77.66 (67.08-85.20)
**FIGO III-IV**			
Urban	248	203	28.54 (22.62-34.71)
rural	215	184	23.38 (17.63-29.63)

Five-year RS was 41% for serous, 31% for other epithelial tumours: HR = 1.37[1.04-1.82], 49% for mucinous and endometrioid: HR = 0.74[0.58-0.96] and 63% for non epithelial cancers: HR = 0.53[0.33-0.84]. Women who lived in urban areas had a better 5-year RS rate compared to those who lived in rural areas: 46% vs. 40% (HR = 0.81[0.67-0.99]).

### Multivariate analyses

Multivariate analyses of RS confirmed that age (p = 0.003), FIGO stage (p < 0.0001), histological type (p = 0.01), place of residence (p = 0.04) and period of diagnosis (p < 0.0001) were independent prognostic factors for RS in ovarian cancer. As compared to age younger than 50 years, age older than 70 years was associated with lower survival HR = 1.60 [1.21-2.12]. An increased risk of death was also observed with advanced stage as compared to stage I with HR = 4.43 [2.63-7.46], HR = 8.80 [5.81-13.34] and HR = 13.07 [8.26-20.66] for FIGO II, III and IV, respectively. As compared to serous type, other epithelial tumours apart from mucinous and endometrioid were associated with lower survival: HR = 1.62[1.22-2.16]. As compared to women who lived in rural areas, women who lived in urban areas had better survival HR 0.82[0.67-0.99] (Table 3).

### Changes in the survival rate over time

During the periods 1982-1989 and 1998-2005, women diagnosed with ovarian cancer had an improvement in 5-year RS rates from 41% to 49%; but no difference in the survival rate was found between the periods 1982-1989 and 1990-1997. Therefore, these two periods were combined for the rest of the analyses. To describe the improvement according to variables of interest over time, RS from 1982 to 1997 was compared with that from 1998 to 2005 (table 5). After dividing our study population into early (stage I-II) and advanced (stage III-IV) stage disease, the improvement seemed to be more pronounced in advanced stage (20% to 36%) and in younger and middle-aged patients.

**Table 5:** Trends in 5-year relative survival rates over time

Variables	5-year relative survival (95%CI)	
	**1982-1997**	**1998-2005**

**Age**		
< 50	55 (44.6-64.3)	65 (50.0-76.4)
50-70	38 (31.9-44.9)	52 (42.1-60.2)
≥ 70	31(22.2-39.8)	33 (22.0-44.8)
**Stage at diagnosis**		
I-II	74 (66.1-80.9)	81 (68.0-89.0)
III-IV	20 (15.2-24.8)	36 (28.7-43.7)
**Histological type**		
Serous	37 (31.0-43.1)	49 (40.4-57.9)
Non serous	40 (32.1-47.5)	49(39.0-58.3)
**Place of residence**		
Urban	43 (36.3-49.2)	51 (42.1-59.9)
Rural	37 (29.9-43.7)	47 (37.0-56.0)

## Discussion

Our study included all cases of invasive ovarian cancer diagnosed between 1982 and 2005 in a well-defined population using a cancer registry specialized in ovarian cancer.

The results of our study showed a progressive decline in the survival rate with 3-year and 5-year RS rates of 55% and 44%, respectively. These results are in agreement with those reported in western Sweden [11] where the 5-year RS rate for patients diagnosed with ovarian cancer from 1993 to 1998 was 46%. In the same way, a population-based study in Germany [12] showed a 5-year RS rate of 45% among patients with ovarian cancer, diagnosed from 1999 to 2003.

In line with the present study, many studies on ovarian cancer found age to be an independent prognostic factor [7,11,13-16]. It is important to emphasize that our analysis was based on a population-based cohort and that it is, to our knowledge, the only one to assess independent prognostic factors in ovarian cancer using RS as the primary end-point. In our study, women aged younger than 50 years had the best prognosis of all age groups. Multivariate relative analyses showed that only patients aged older than 70 years had significantly worse survival as compared to those younger than 50 years. This can be due to the particular management of ovarian cancer in elderly patients who are less likely to receive standard combination therapy [17,18], and thus benefit less from advances in treatment.

As in other studies [11,13-16,19], the results of the present study showed clearly that survival is stage-dependent. In fact, multivariate analyses showed that the FIGO stage was the most powerful prognostic factor, with a hazard ratio of 13 in stage IV in the multivariate analysis. In our study, we found 5-year RS rates of 85%, 48%, 29% and 14% for stages I, II, III and IV, respectively. This could be compared with the findings obtained from the SEER program which showed 5-year RS rates of 88.3%, 65.0%, 34.1% and 19.7%, respectively for patients with stage I, II, III and IV ovarian cancer diagnosed between 1988 and 2001[16]. The results were closer for stage I, III and IV, but a considerably lower survival rate was recorded in our sample for stage II disease. This difference concerning stage II may be explained by disparities in the proportion of patients who did not receive optimal debulking and were thereby understaged. It is worth noting that recent studies [20,21] showed that a large number of ovarian cancer patients with clinically localized disease still underwent incomplete surgical staging.

Unlike some studies [11,13] the result of the present study found histological subtype to be an independent prognostic factor in ovarian cancer. Our results showed that patients with non-epithelial or endometrioid-mucinous tumours had better survival as compared to those with serous tumours in univariate analyses of RS but not in the multivariate analyses. Moreover, patients with other epithelial subtypes, mainly clear cell and unspecified adenocarcinoma tumours, were found to have worse survival as compared to those with serous tumours. Clear cell histology is generally accepted as an unfavourable histology, but the results concerning other histology subtypes are contradictory. In a recent study [14] comparing the survival of women with clear cell versus other epithelial ovarian cancers, clear cell had poorer overall survival. In a recent review [7], clear cell histology and mucinous cancers were associated with worse survival. In contrast, an Australian study [15] found that endometrioid and non-epithelial tumours were associated with better survival as compared with serous tumours, while non-specified adenocarcinoma correlated with worse survival.

In multivariate analyses, the place of residence was an independent prognostic factor for relative survival in ovarian cancer. Women who lived in rural areas had a greater excess risk of death due to ovarian cancer than did those who lived in urban areas. This may be due to more restricted access to high performance treatment facilities in these regions. The results of the multivariate model suggest that differences in stage distribution (that women in rural areas may be diagnosed at a later stage) do not explain the poorer survival in rural areas, as the hazard ratio remained unchanged in the multivariate model.

In our study, we found the period of diagnosis to be an important independent prognostic factor. Patients diagnosed during the period 1998-2005 had significantly better survival as compared to those diagnosed during the period 1982-1989, whereas no difference in survival was found for women diagnosed in the period 1990-1997. Similar results were found by Engel et al in a population-based cohort of ovarian cancer patients during 1978-1997. This study [5] found that survival rates before and after 1988 were similar.

During the study period, we found an improvement in RS in women with early stage as well as in those with advanced stage ovarian cancer, but the improvement was more pronounced for women with advanced stages (Table 5). This may be explained by the importance of maximal cytoreduction in the recent period and by the fact that the workup and staging are more comprehensive. In fact, in our study, the proportion of FIGO stage III was higher in the most recent period compared to the first period of diagnosis (Table 2). In the same way, in their study [22], Chan et al showed a significant improvement across time in the survival of women with surgically stage III and IV diseases.

The survival improvement appearing after 1998 may be attributed to advances in treatment. Several studies have explored the role of surgery in advanced ovarian cancer [23]. Most of these studies supported the role of tumour debulking as the best first-line treatment in women with advanced ovarian cancer. It is also likely that advances in chemotherapy as well as in general medical support have contributed to this improvement.

The strength of the present study is that it includes all patients with ovarian cancer diagnosed from 1982 to 2005 in a well-defined geographical area. Furthermore, the follow up for vital status was nearly complete with a low lost-to-follow-up rate (7%). With this large panel, the results of our study could be considered representative of survival in French patients during this period. Unlike clinical trials, in which patients are highly selected, population-based survival studies are based on heterogeneous groups and can be used to determine prognostic factors in cancer, with little potential for selection bias [24].

## Conclusion

The results of this population-based study showed that advanced age at diagnosis, advanced FIGO stage, period of diagnosis, histological type and place of residence are independent prognostic factors in ovarian cancer and that long-term survival has improved over the last 10 years. Despite the overall improvement in ovarian cancer survival, novel treatment strategies are still warranted.

## Competing interests

The authors declare that they have no competing interests.

## Authors' contributions

ZH, SC, PA, FB conceived and designed the study, ZH, SC, PR, TA, MLP, LA participated in the quality control and data acquisition, ZH, PA, FB, TSD, JG performed statistical analysis, data interpretation and wrote the manuscript. All authors read and approved the final manuscript.

## Pre-publication history

The pre-publication history for this paper can be accessed here:

http://www.biomedcentral.com/1471-2407/10/622/prepub
